# Soluble (Pro)Renin Receptor Levels Are Regulated by Plasma Renin Activity and Correlated with Edema in Mice and Humans with HFrEF

**DOI:** 10.3390/biomedicines10081874

**Published:** 2022-08-03

**Authors:** Inna P. Gladysheva, Ryan D. Sullivan, Kodangudi Ramanathan, Guy L. Reed

**Affiliations:** 1 Department of Medicine, University of Arizona College of Medicine-Phoenix, Phoenix, AZ 85004, USA; ryansullivan@arizona.edu (R.D.S.); guyreed@arizona.edu (G.L.R.); 2 Memphis Veterans Affairs Medical Center, Memphis, TN 38163, USA; kramanat@uthsc.edu

**Keywords:** soluble (pro)renin receptor, renin plasma activity, HFrEF, edema

## Abstract

Symptomatic heart failure with reduced ejection fraction (HFrEF) is characterized by edema and chronic pathological activation of the classical renin–angiotensin–aldosterone system (RAAS). The soluble (pro)renin receptor (s(P)RR) is released into circulation by proteolytic cleavage of tissue expressed (P)RR and is a candidate biomarker of RAAS activation. However, previous studies linked elevated levels of s(P)RR in patients with HFrEF to renal dysfunction. Utilizing prospectively enrolled patients with comparable rEF, we show that increased plasma levels of s(P)RR are associated with symptomatic HF (characterized by edema), independent of chronic renal dysfunction. We also found that s(P)RR levels were positively correlated with patient plasma renin activity (PRA). Normotensive mice with dilated cardiomyopathy (DCM) and HFrEF, without renal dysfunction, showed plasma s(P)RR and PRA patterns similar to human HFrEF patients. Plasma s(P)RR levels positively correlated with PRA and systemic edema, but not with EF, resembling findings in patients with HFrEF without chronic kidney dysfunction. In female DCM mice with elevated PRA levels and plasma s(P)RR levels, a randomized, blinded trial comparing the direct renin inhibitor, aliskiren vs. vehicle control, showed that direct renin inhibition normalized PRA, lowered s(P)RR, and prevented symptomatic HFrEF. Considered in light of previous findings, these data suggest that, in HFrEF, in the absence of renal dysfunction, elevation of plasma s(P)RR levels is caused by increased PRA and associated with the development of systemic edema.

## 1. Introduction

Dilated cardiomyopathy (DCM), one major cause of heart failure (HF), is characterized by progressive heart enlargement with a reduced ejection fraction (rEF) [1,2,3]. Despite the best available therapies [4,5,6,7], for most patients, DCM progresses relentlessly from at-risk status (or stage A HF) to pre-HF (stage B HF) and then to symptomatic HF (sHF, stages C–D) with progressive fluid–salt retention (edema), cachexia/sarcopenia, disability, and a 50% mortality rate within 5 years of diagnosis [1,8,9,10]. New diagnostic and therapeutic strategies are needed to block the progression of DCM to sHF, prolong and improve the quality of life.

sHFrEF is characterized by the chronic pathological activation of the classical renin–angiotensin–aldosterone system (RAAS), dysregulation of the protective RAAS arm (Ang 1–7/MAS), and impairment of the natriuretic peptide (NP) system, which promotes salt and water retention leading to clinical HF signs and symptoms from edema, a decline in the quality of life, and premature death [9,11,12,13,14,15,16,17,18,19].

Systemic RAAS activation occurs with increased plasma renin activity (PRA), which initiates the first and rate-limiting step [18]. Circulating active renin and pro-renin bind to transmembrane (pro)renin receptor (P)RR to activate local tissue classical RAAS or initiate independent intracellular signaling [20]. The physiological role of (P)RR, among others, includes regulation of fluid balance; it is pathologically linked to renal and cardiovascular dysfunction including HF [21,22,23]. Cleavage of tissue expressed (P)RR by the serine protease furin [24] and/or metallopeptidase ADAM19 [25] leads to intercellular release of soluble (P)RR (s(P)RR) followed by its release into circulation. The circulating level of s(P)RR is suggested to be a surrogate biomarker of tissue RAAS activity [26] with a potential functional role in nonproteolytic pro-renin activation [27,28].

High serum s(P)RR levels were associated with HF progression in hemodialysis patients [29] and patients with chronic HF regardless of renal function [30]. In other studies, elevated circulating s(P)RR levels in patients with chronic HF were associated with renal dysfunction [31,32,33]. It remains unclear in HFrEF if s(P)RR levels reflect only renal dysfunction or the severity of systolic dysfunction and/or edema (a hallmark of sHF). A functional relationship between circulating s(P)RR and PRA levels in HFrEF also remains undetermined. Here, we report clinical and preclinical data demonstrating that, in DCM-HFrEF patients and mice with or without sHF, and without chronic kidney disease (CKD), elevated plasma levels of s(P)RR are associated with edema and potentially causally linked to increased PRA.

## 2. Materials and Methods

### 2.1. Study Population

Three groups of patients (*n* = 16 per group, ages 50–70) admitted to the medical service of the Memphis Veterans Affairs Medical Center (VAMC) were prospectively enrolled as previously described: (1) no HF and normal ejection fraction (healthy, mean EF: 63% ± 3%); (2) no HF and reduced EF (DCM, mean rEF: 27% ± 7%), and sHF with rEF (DCM-sHF, mean rEF: 24% ± 8%) [17]. The inclusion and exclusion criteria were previously described in detail [17]. Patient groups were comparable in terms of age, sex, and race. All patients were male, reflecting the VAMC patient population. The use of medications such as aspirin, clopidogrel, beta-blockers, angiotensin-converting enzyme inhibitors, angiotensin receptor blockers, warfarin, nitrates, statins, and antiarrhythmic agents were similar between the enrolled groups (*p* > 0.1). Patients with CKD (estimated glomerular filtration rate, eGFR < 60 mL/min/1.73 m^2^), pulmonary hypertension, myocardial infarction within the last 6 weeks, critical valvular heart disease, metastatic or terminal cancer, morbid obesity (BMI > 35), and cardiopulmonary support were excluded from the study. sHF was defined by abbreviated Framingham criteria, including peripheral edema, and by plasma level of a B-type natriuretic peptide, BNP > 400 pg/mL. There was no significant difference in EF between groups with rEF regardless of sHF status. The selected group sample size (based on our previous study [13]) allowed us to detect the difference in targeted plasma markers levels between enrolled groups of patients with statistical power >90%. The study was approved by the Institutional Review Board of the Memphis VAMC and conformed to the principles and ethical guidelines of the Declaration of Helsinki (1964). All enrolled patients provided their signed informed consent to participate in the study [17].

### 2.2. Mouse Model of DCM-HFrEF

This study utilized the previously established preclinical normotensive mouse model of progressive DCM with preserved kidney function (on C57BL/6J background), generated by cardiac-specific, dominant negative, transgenic suppression of CREB phosphorylation driven by the alpha myosin heavy chain promoter [19,34,35,36,37,38,39,40,41,42]. DCM progression in this model is not associated with cardiac arrhythmias, blood pressure alteration, or kidney failure [19,39].

Experimental mouse studies were conducted in accordance with National Institute of Health (NIH) Guide for the Care and Use of Laboratory Animals and with the Animal Research: Reporting In Vivo Experiments (ARRIVE) guidelines. Mice were housed in an individually ventilated caging system and fed an ad libitum maintenance diet (Envigo Tekland 7912, Madison, WI, USA) within AAALACi accredited facilities under a 12 h/12 h light/dark cycle. Mouse health, behavioral changes including loss of ambulatory activity (fatigue, lethargy, dyspnea, body tremors, and self-isolation), and death records were monitored by investigators or animal care staff daily as we previously reported [19]. Female mice with DCM were randomly and blindly treated with DRI aliskiren hemifumarate (100 mg/kg/day in drinking water; BOC Sciences, Shirley, NY, USA) or vehicle starting from the stage B HF corresponding to 7 weeks of age [40]), and characterized by pathologically elevated PRA levels as we previously reported [39]. Groups were analyzed at the point when vehicle-treated DCM mice progressed to stage D HF, corresponding to ~13 weeks of age [39]. The physiological outcomes were analyzed using clinically relevant diagnostic modalities for translational relevance. The subgroups of mice were sacrificed at 13 weeks of age for organ and plasma collection after systolic function (EF) and systemic edema (extracellular water, ECW) assessed by quantitative magnetic resonance (QMR) were recorded.

Studies were approved by the Institutional Animal Care and Use Committees at the University of Arizona College of Medicine–Phoenix (Protocol 17-303, approved 11 December 2017) or the University of Tennessee Health Science Center (Protocol 15-050.0, approved 9 July 2015; Protocol 17-059.0, approved 26 July 2017).

### 2.3. Blood Sample Collection

Venous blood samples of enrolled VAMC patients were collected in standard EDTA-aprotinin tubes to prevent coagulation and proteolysis of targeted proteins and immediately stored on ice [17]. Mouse blood samples were collected by cardiocentesis with syringes supplied with EDTA (Sigma-Aldrich, MO, USA) and aprotinin (Sigma-Aldrich, MO, USA) as previously described [40]. The blood samples were centrifuged at 4 °C, 3000× *g* for 20 min, and the plasma samples were aliquoted and stored at −80 °C until analyzed.

### 2.4. Plasma Biomarker Measurements

Plasma levels of s(P)RR were measured by solid-phase sandwich enzyme-linked immunosorbent assay (ELISA) using IBL Immuno-Biological Labs Co. Ltd., Fujioka-Shi, Japan kit (Code number 27782) for human and mouse plasma samples. The proper dilution factors and the optimum incubation time with test samples were determined in the laboratory.

Renin enzymatic activity in plasma samples (or plasma renin activity, PRA) was measured and quantified by cleavage of the fluorescence resonance transfer (FRET) peptide substrates optimized for human renin or mouse renin with minimal background autofluorescence, FRET-QXL™520/5-FAM, using the SensoLyte 520 renin assay kits (AnaSpec, Fremont, CA, USA) as previously reported [18,19,39,40,41].

### 2.5. Extracellular Water Analysis

Systemic extracellular water (ECW) or free water, was objectively recorded by the QMR system (EchoMRI 4-in-1 Analyzer, Echo Medical Systems, Houston, TX, USA) as we previously described [19,40,41]. The fully conscious mice (after urination) were noninvasively examined (~1.5 min recording time) at 13 weeks, prior to necropsy.

### 2.6. Echocardiography

Systolic function and heart rate were assessed in anesthetized mice by transthoracic echocardiography (Vevo 2100/3100 Imaging Systems, VisualSonics, Toronto, ON, Canada) as we described [19,38,39,40,41,42]. Mice were maintained at an anesthetized heart rate of 450 ± 50 beats per minute and 37 ± 1 °C rectal temperature. Data were blindly analyzed by Vevo LAB 3.1.0 software using company standard protocols for systolic function.

### 2.7. Statistical Analysis

Data were analyzed with appropriate parametric or nonparametric methods with GraphPad Prism 9.0 software (GraphPad Software, San Diego, CA, USA) using Mann–Whitney multiple comparison between groups, one-way ANOVA, or two-way ANOVA with Tukey’s multiple comparison test and Pearson or Spearman correlations. Data are presented as the mean ± SE. A two-tailed *p*-value < 0.05 was considered statistically significant and denoted in the figure legends as * *p* < 0.05, ** *p* < 0.01, *** *p* < 0.001, and **** *p* < 0.0001.

## 3. Results

### 3.1. Plasma s(P)RR Levels Elevate as Clinical HFrEF Progress and Positively Correlate with PRA

**Figure 1:**
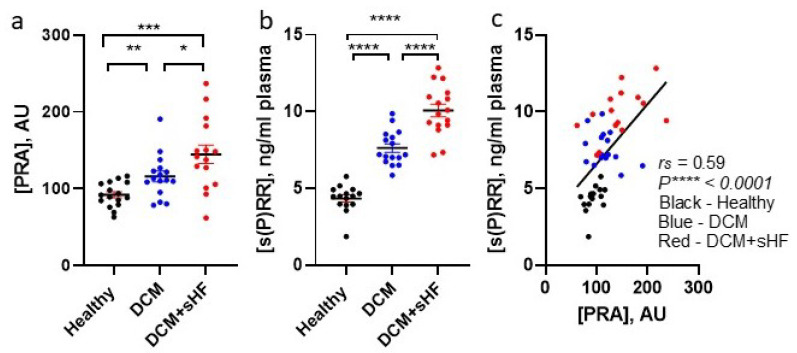
Plasma renin activity (PRA) [18] (**a**) and soluble (pro)renin receptor, s(P)RR (**b**) levels alteration in clinical DCM and symptomatic HFrEF. (**c**) Spearman correlation of PRA vs. s(P)RR. Arbitrary units (AU). Comparisons between groups were analyzed with Mann–Whitney multiple comparison; * *p* < 0.05, ** *p* < 0.01, *** *p* < 0.001, and **** *p* < 0.0001. Black: healthy—no HF and normal EF; Blue: DCM (dilated cardiomyopathy)—pre-symptomatic HFrEF; Red: DCM + sHF (HF symptoms)—symptomatic HFrEF.

We measured plasma s(P)RR levels and correlated them with PRA levels (Figure 1) in three groups of prospectively enrolled VMAC patients without CKD (eGFR > 60 mL/min/1.73 m^2^; *n* = 16/group) [17]. The groups included patients without systolic dysfunction and HF (healthy group), no HF with rEF (DCM group), and sHF with rEF (DCM + sHF group). Systolic dysfunction (rEF) was comparable between DCM groups. This experimental clinical setting allowed us to investigate whether systolic dysfunction and HF signs, including edema, modulate s(P)RR independently from kidney dysfunction. Both PRA and s(P)RR plasma levels were significantly increased in clinical pre-symptomatic HF with rEF (DCM group) vs. the healthy group. Both levels were further elevated in the DCM + sHF group, which was characterized by the onset of edema and other Framingham HF diagnostic criteria, (Figure 1a,b). Spearman’s analysis demonstrated that s(P)RR levels are positively correlated with PRA levels (*r*_s_ = 0.59, *p* < 0.0001; Figure 1c). Collectively, these data illustrate that, in DCM patients without CKD, the positive association between s(P)RR and PRA levels positively aligned with the presence of edema, thus reflecting the degree of sHF.

### 3.2. Association between Plasma s(P)RR and PRA Levels in DCM-HFrEF Mouse Model

**Figure 2:**
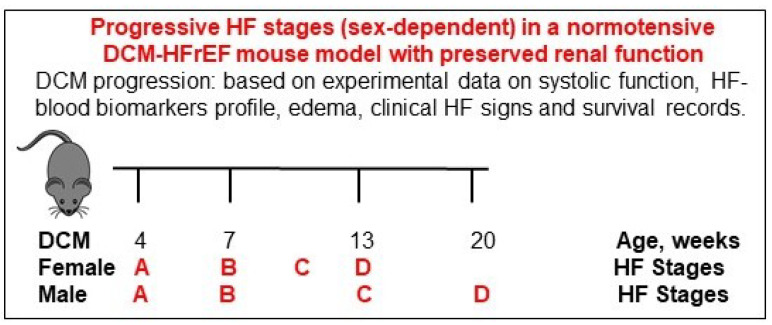
Schematic presentation of established progressive mouse model of dilated cardiomyopathy heart failure with reduced ejection fraction (DCM-HFrEF).

We and others have extensively characterized a mouse model of DCM-HFrEF, without kidney dysfunction, with high translational relevance to human DCM-HFrEF [19,34,35,36,37,38,39,40,41,42]. In these mice, we described the longitudinal progression of HF stages according to systolic dysfunction, cardiac remodeling and fibrosis, edema, visible HF signs (fatigue, dyspnea, etc.), cachexia/sarcopenia, well-defined HF biomarker profiles, and premature death [19,38,39,40]. The DCM-HFrEF mice recapitulate human HF progressive stages [8,9] in an age- and sex-related manner, from at risk for HF (stage A) to pre-HF (stage B: to progressive decline in contractile function (rEF) and increasing heart dilation), HF (stage C: systemic and pulmonary edema with increases in HF biomarkers (NT-pro-ANP and BNP/NT-pro-BNP, etc.) and cardiac fibrosis), and advanced HF (stage D: edema, pulmonary effusion, visible HF signs, decreased muscle/fat mass (cachexia/sarcopenia), with a median survival age of 13–14 weeks in females and 20 weeks in males) (Figure 2).

**Figure 3:**
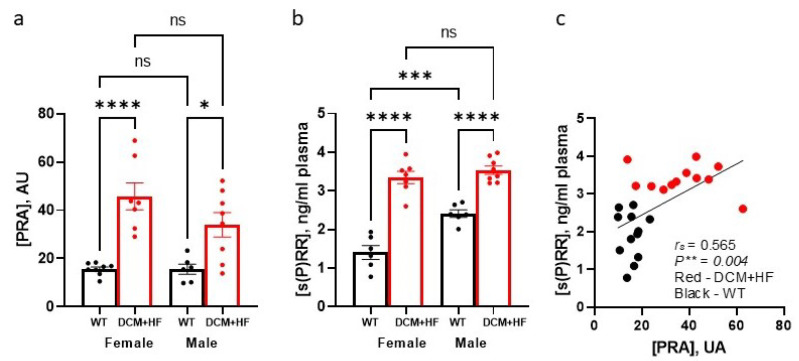
Levels of plasma renin activity (PRA) and soluble (pro)renin receptor (s(P)RR in male and female control mice vs. DCM mice with stage D heart failure: (**a**) levels of PRA; (**b**) plasma levels of (s(P)RR; (**c**) Spearman correlation analysis of combined s(P)RR (**c**) vs. combined PRA. Groups represent female mice with DCM (13 weeks of age, corresponding stage D HF, DCM + HF) and congenic wild-type (WT) mice at 13 weeks of age, and male mice with DCM (20 weeks of age corresponding to stage D HF) and congenic WT at 20 weeks of age. Arbitrary units (AU). Data were analyzed with two-way ANOVA with Tukey’s multiple comparison; * *p* < 0.05, ** *p* < 0.01, *** *p* < 0.001, and **** *p* < 0.0001; ns—not significant. HF—heart failure; DCM—dilated cardiomyopathy.

Like humans with DCM-HFrEF, both sexes of mice with DCM-HFrEF have progressive pathologically elevated PRA levels, activation of classical RAAS, and an impaired protective RAAS arm [19,39,40]. We analyzed plasma levels of PRA and s(P)RR in female and male DCM mice at the age corresponding to advanced HF (stage D HF) vs. congenic WT mice (Figure 3 a,b). Data demonstrate that advanced HF is characterized by significant elevation of both PRA and s(P)RR levels in both sexes. Spearman’s analysis of combined male and female mouse groups demonstrated that plasma s(P)RR levels are positively correlated with PRA levels (*r*_s_ = 0.57, *p* < 0.004) signifying a significant association of s(P)RR with HF (Figure 3c).

### 3.3. Normalization of PRA with Direct Renin Inhibitor Normalizes Plasma s(P)RR Levels in DCM-HFrEF Mice

**Figure 4:**
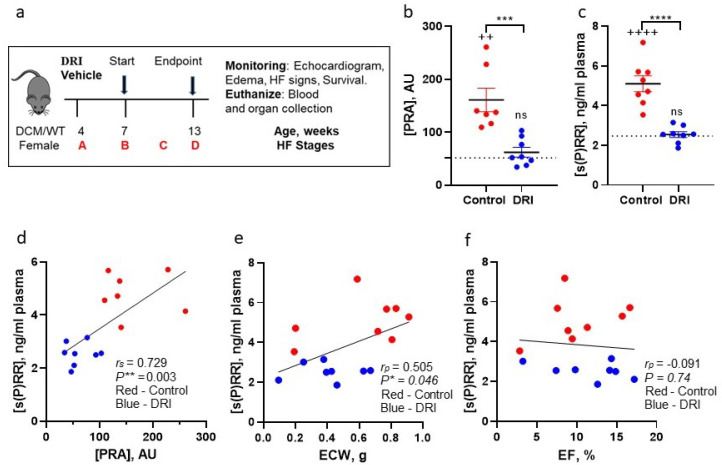
Plasma soluble (pro)renin receptor (s(P)RR) levels are correlated with plasma renin activity (PRA) and edema and suppressed by direct inhibition of renin activity in mice with DCM. (**a**) Experimental design: female mice with DCM (7 weeks of age corresponding stage B HF) were randomly assigned to treatment with direct renin inhibitor (DRI) aliskiren (DRI) in drinking water or vehicle (control, plain drinking water). PRA [40] (**b**) and s(P)RR (**c**) level alterations in mice with DCM at 13 weeks of age after treatment with vehicle (control) or DRI. Arbitrary units (AU). Data were analyzed with one-way ANOVA with Tukey’s multiple comparison. (**d**) Spearman correlation analysis of s(P)RR (**b**) vs. PRA (**a**). The values for the wildtype (WT, *n* = 4 or 7) are shown as a black dotted line for reference. Association of s(P)RR plasma levels with systemic edema assessed by ECW (**e**) or EF (**f**) by Pearson’s correlation analysis. QMR—quantitative magnetic resonance for the extracellular water retention (ECW) monitoring; EF—ejection fraction. Data are presented as the mean ± SE; * *p* < 0.05, ** *p* < 0.01, *** *p* < 0.001 and **** *p* < 0.0001 for control vs. DRI; ^++^ *p* < 0.01 and ^++++^ *p* < 0.0001 for control vs. WT; ns—not significant.

To examine the functional relationship between plasma s(P)RR and PRA during DCM progression to symptomatic HFrEF, we analyzed and correlated their levels in groups of DCM female littermate mice enrolled in a randomized, blinded preclinical trial that compared outcomes of treatments with DRI aliskiren vs. vehicle control [40]; the wildtype (WT) congenic group was used for control (Figure 4a). We reported that normalization of PRA levels (equivalent to WT group) in DCM mice treated with aliskiren, in comparison to control DCM mice treated with vehicle (Figure 4b), improved systolic function (EF, *p* < 0.05; cardiac output, *p* < 0.01), reduced systemic edema (ECW levels, *p* < 0.0001), and prolonged life (*p* < 0.05) [40]. Treatment with the DRI vs. vehicle significantly reduced plasma s(P)RR to levels detected in the WT control group (*p* < 0.0001, Figure 4c). Plasma s(P)RR and PRA levels were significantly correlated (*r*_s_ = 0.73, *p* < 0.0001; Figure 4d). The levels were higher in the DCM group of mice treated with vehicle (red dots) that progressed to HF at 13 weeks of age (defined by systemic edema assessed by QMR) than in the DCM group treated with DRI (blue dots) and characterized by the absence of HF [40]. Consistently, s(P)RR levels were positively correlated with ECW (Figure 4e), indicating that s(P)RR levels are associated with systemic edema. The plasma levels of s(P)RR were not associated with EF (Figure 4f), suggesting that systolic function does not affect receptor levels in circulation.

## 4. Discussion

Chronic renal dysfunction (eGFR ≤ 60 mL/min/1.73 m^2^) accelerates HFrEF decompensation and mortality [43,44,45]. In patients with sHF with rEF, the elevation of circulating s(P)RR levels was associated with renal dysfunction [31,32,33]. In contrast, in this study, we demonstrated that an elevation in the circulating level of s(P)RR in DCM patients reflected the progression of systolic dysfunction to symptomatic HFrEF, characterized by edema independent of CKD. Moreover, s(P)RR levels are positively correlated with PRA levels. The limited patient group size may have reduced our power to detect a linkage between the presence of HF-related edema and plasma s(P)RR levels or a s(P)RR level correlation with PRA. Normotensive DCM mice of both sexes, which mirror human DCM progression and transition to symptomatic HfrEF without kidney dysfunction [19,36,38,39,40], demonstrated the same patterns of circulating s(P)RR and PRA. Our data showed that, in female mice with DCM, treatment with direct renin inhibitor (DRI), aliskiren, not only normalized the elevated pathological PRA and attenuated progression to HFrEF [40], but also simultaneously reduced plasma s(P)RR levels to that of WT congenic mice.

Classical RAAS activation is thought to compensate for impaired cardiac function through increased salt and water retention and other actions. Yet, chronic overactivation of RAAS by PRA has deleterious effects on cardiac structure and performance, leading to symptomatic HFrEF characterized by edema [16,18]. Translational and clinical evidence suggests that as DCM progresses, pathologically elevated PRA enhances systolic dysfunction, promotes edema, and accelerates the development of symptomatic HFrEF [39,40,46]. The presented data demonstrate that in humans and mice with DCM-HFrEF without renal impairment, a significant rise in plasma s(P)RR is associated with the presence of edema and positively correlated with PRA levels. The correlation between circulating levels of s(P)RR and PRA may be unique for HFrEF without CKD, as it has not been observed in patients without HF or those with CKD. Thus, there was no correlation between s(P)RR and active renin or pro-renin in healthy subjects and non-HF patients with an overactivated RAAS [47]. s(P)RR levels were not correlated with PRA and HF stage in patients with severe HFrEF and kidney failure (eGFR in a range of about 60–15 mL/min/1.73 m^2^) [33]. Additionally, there was no correlation between s(P)RR and PRA serum levels in patients with autosomal dominant polycystic kidney disease without HF [48].

Furthermore, we investigated the functional association between circulating s(P)RR and PRA in DCM mice as they progress from pre-symptomatic stage B HF to advanced symptomatic HFrEF (stage D), characterized, as we previously reported [39,40], by systemic edema, cachexia/sarcopenia, and premature mortality. These mice are normotensive and have preserved renal function, eliminating confounding outcomes from these variables. The randomized, blinded trial demonstrated improvement of systolic function, prevention of systemic edema (symptomatic HFrEF), and improved survival in the DCM mouse group treated with DRI aliskiren vs. DCM littermate control mice treated with vehicle [40,49]. DRI treatment normalized pathologically elevated PRA and plasma s(P)RR levels to levels detected in the congenic mouse group without DCM. The positive association of plasma s(P)RR levels with systemic edema agrees with the previously reported antidiuretic role of urine s(P)RR in regulating fluid homeostasis and urine concentrating capability in rodents [50,51]. Nevertheless, it remains to be investigated whether, in DCM-HFrEF without CKD, elevated circulating s(P)RR has a causative effect or is simply correlated with HF and edema.

Little is known about the role of circulating s(P)RR in pro-renin activation in physiological and pathological conditions [18,27,28]. As DCM progressed to sHF, circulating s(P)RR might bind and nonproteolytically activate pro-renin, similar to tissue-expressed (P)RR [20], contributing to pathologically increased PRA levels directly associated with edema [40] and conversion of angiotensinogen to angiotensin I.

Cardiac (P)RR transcript (ATP6AP2 gene) and protein expression were significantly elevated in patients with idiopathic DCM and NYHA class II–IV compared to healthy donor hearts [52] and in rodent models of HFrEF [52,53,54]. Preclinical studies in mice demonstrated that pathologically expressed (P)RR contributed to developing DCM and HFrEF [55,56,57,58,59,60]. Aliskiren treatment, at a dose of 10 mg/kg per day via osmotic mini-pump for 6 weeks, reduced cardiac transcripts and protein levels of (P)RR in TGR(mRen2)-27 rats with streptozotocin-induced diabetic cardiomyopathy with diastolic dysfunction in the absence of edema [61]. In this rat model, aliskiren treatment (10 or 30 mg/kg per day, 10 weeks i.v.) also reduced (P)RR expression in the renal compartments [62]. These data support the finding of our study that lowering PRA affects circulating levels of sP(RR). The reduced plasma levels of s(P)RR in DRI-treated DCM mice may be due in part to the diminished gene and protein expression or altered processing/cleavage of (P)RR.

## 5. Conclusions

**Figure 5:**
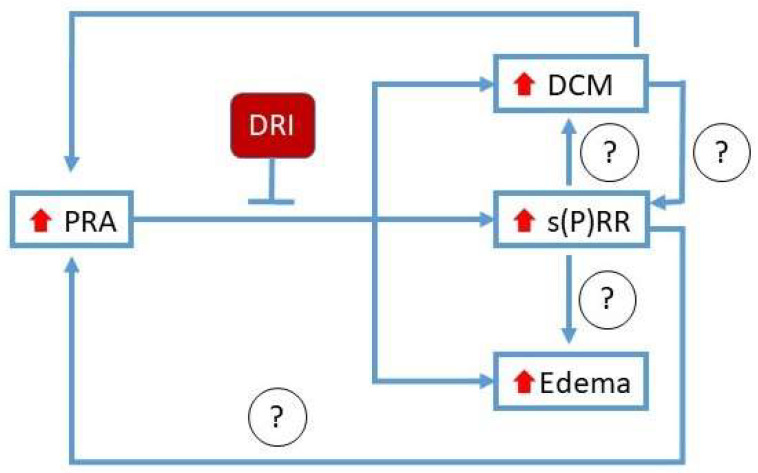
Simplified schematic summary. Levels of PRA, s(P)RR, and edema increase in worsening dilated cardiomyopathy (DCM) with HF without renal impairment. Levels of PRA and s(P)RR are closely correlated. Increased PRA activates the RAAS to enhance edema formation and worsen DCM. Normalization of PRA level with a direct renin inhibitor (DRI) suppresses s(P)RR, edema, and progression of cardiomyopathy to HFrEF. Increased levels of s(P)RR may worsen DCM and promote edema through direct effects or through indirect effects, by increasing PRA. In HFrEF without chronic kidney disease, the protective effects of DRI may be mediated in part through suppression of s(P)RR.

Taken together, these DCM-HFrEF clinical and preclinical results indicate that plasma s(P)RR levels are significantly correlated with PRA and the presence of edema/congestion or symptomatic HF. In both humans and mice, elevated levels of circulating s(P)RR may be a biomarker (and potential regulator) of pathological fluid retention of HF origin, in the absence of chronic kidney dysfunction. Experimental normalization of PRA by the DRI, aliskiren, in normotensive mice with DCM without renal dysfunction attenuates progression to HFrEF and decreases levels of circulating s(P)RR. This may be due to downregulation of tissue expression, as well as other mechanisms that are yet to be investigated. Further research into the functional role of circulating s(P)RR in DCM and HFrEF is needed to support its role as a potential therapeutic target. Clinical studies are required to confirm that DRIs reduce s(P)RR circulating levels in patients with HFrEF without CKD. The potential mechanisms through which DRI might affect s(P)RR levels in circulation and circulating s(P)RR might contribute to edema promotion in HFrEF are schematically summarized in Figure 5.

## 6. Patents

UA Tech Launch, along with the authors, have filed USA patents application related to (1) the methods of personalized treatment for cardiomyopathy and heart failure and other related diseases by measuring renin activity, pro-renin and (pro)renin receptor levels in blood (Pub. No.: 20210262008, 26 August 2021), and (2) extracellular water analysis as a measure of edema (Pub. No.: US 20210263120, 26 August 2021).

## Data Availability

Data generated for this study are available upon reasonable request to the corresponding author I.P.G.
